# Revision of the genus *Ptomaphagus* Hellwig from eastern Asia (Coleoptera, Leiodidae, Cholevinae)

**DOI:** 10.3897/zookeys.715.20497

**Published:** 2017-11-14

**Authors:** Cheng-Bin Wang, Michel Perreau, Jan Růžička, Masaaki Nishikawa

**Affiliations:** 1 Department of Ecology, Faculty of Environmental Sciences, Czech University of Life Sciences Prague, Kamýcká 129, CZ-165 21 Praha 6, Czech Republic; 2 IUT Paris Diderot, Université Paris Diderot, Sorbonne Paris Cité, case 7139, 5 rue Thomas Mann, F-75205 Paris cedex 13, France; 3 Kashiwagaya 1112-16, Ebina, 243-0402 Japan

**Keywords:** Cholevinae, eastern Asia, Leiodidae, new species, *Ptomaphagus*, species group, taxonomy

## Abstract

The species belonging to the genus *Ptomaphagus* Hellwig, 1795 (Coleoptera, Leiodidae, Cholevinae, Ptomaphagini) from eastern Asia are assigned to three species groups. Group *yasutoshii* has a single species: P. (s. str.) yasutoshii Nishikawa, 1993 from Taiwan, China. Group *nepalensis* with three species: P. (s. str.) nepalensis Perreau, 1988 from Nepal and P. (s. str.) masumotoi Nishikawa, 2011 from Thailand are redescribed, and P. (s. str.) piccoloi Wang, Růžička, Nishikawa, Perreau & Hayashi, 2016 is recorded for the first time from China (Zhejiang). Group *sibiricus* with seven species, including two newly described Chinese ones P. (s. str.) funiu **sp. n.** from Henan, and P. (s. str.) haba **sp. n.** from Yunnan, and five known species: P. (s. str.) chenggongi Wang, Nishikawa, Perreau, Růžička & Hayashi, 2016, P. (s. str.) hayashii Wang, Růžička, Perreau, Nishikawa & Park, 2016, P. (s. str.) kuntzeni Sokolowski, 1957 (distribution records from Myanmar excluded), P. (s. str.) sibiricus Jeannel, 1934 and P. (s. str.) tingtingtae Wang, Nishikawa, Perreau, Růžička & Hayashi, 2016. Specimens of other undescribed species of the group *sibiricus* are also recorded, revealing a high diversity of this genus in eastern Asia, especially in central and north Sichuan, China, which essentially remains to be investigated. Relevant morphological characters of the examined species are illustrated with colour plates, and their known distributions are mapped. A key to species of *Ptomaphagus* from eastern Asia is provided.

## Introduction

The genus *Ptomaphagus* belongs to the subtribe Ptomaphagina of the tribe Ptomaphagini (Leiodidae, Cholevinae) and was introduced by Hellwig (1795) based on a single species *Tritoma sericea* Fabricius, 1787 (= *Silpha subvillosa* Goeze, 1777) from Europe, which was fixed as the type species of the genus by monotypy. It is the most speciose genus (including 138 known species worldwide) in the tribe Ptomaphagini. However, the nominotypical subgenus, which is limited to the Palaearctic and north Oriental Regions has only 30 species (Perreau 2000, Nishikawa 2011, Wang et al. 2016a, 2016b, 2016c).

Considering the fauna of China, only four representatives of the subgenus Ptomaphagus s. str. had been recorded from Taiwan Island, two of which were just recently described in a previous paper in this series (Wang et al. 2016b). For the vast mainland of China, there were no records of this genus before this study.

In this paper, two new species are described: Ptomaphagus (s. str.) funiu sp. n. from Henan Province, China and P. (s. str.) haba sp. n. from Yunnan Province, China. P. (s. str.) piccoloi Wang, Růžička, Nishikawa, Perreau & Hayashi, 2016 is recorded for the first time from China (Zhejiang Province). Several unidentified *Ptomaphagus* species from central and north Sichuan Province are discussed here also, without descriptions due to the limited number of available specimens. This reveals a high diversity in this region. Moreover, P. (s. str.) nepalensis Perreau, 1988 from Nepal and P. (s. str.) masumotoi Nishikawa, 2011 from Thailand are redescribed, and record of P. (s. str.) kuntzeni Sokolowski, 1957 from Myanmar is discounted. Relevant morphological characters of the examined species are illustrated with colour plates, and their known distributions are mapped. All species from eastern Asia are assigned to one of three species groups, and a key to all the investigated taxa is provided.

## Materials and methods

Specimens were relaxed and softened in a hot saturated solution of potassium hydroxide for 4 minutes (for mounted dry specimens) or 8 minutes (for alcohol-preserved specimens), and then transferred to distilled water to rinse the residual potassium hydroxide off and stop any further bleaching. The softened specimens were moved into glycerine and dissected there to observe morphological details. After examination, the body parts were mounted on a glass coverslip with Euparal Mounting Medium for future studies. Habitus photographs were taken using a Canon macro photo lens MP-E 65mm on a Canon 550D. Observations, photographs, and measurements of morphological details were performed using an Olympus BX53 microscope with an Olympus DP73 camera. The final deep focus images were created with Zerene Stacker 1.04 stacking software. Adobe Photoshop CS6 was used for post-processing. Exact label data are cited, while authors’ remarks and addenda are placed in square brackets; separate label lines are indicated by a slash (/), and separate labels are indicated by a double slash (//). Measurements are averages taken from 5 specimens.

The material examined for this study is deposited in the following collections and museums (with names of curators in parentheses):

**BMNH** Natural History Museum (formerly British Museum), London, United Kingdom (M. Barclay)

**CAPE** Collection of Andreas Pütz, Eisenhüttenstadt, Germany

**CCBW** Collection of Cheng-Bin Wang, Chengdu, China

**CJRZ** Collection of Jan Růžička, Prague, Czech Republic

**CMNE** Collection of Masaaki Nishikawa, Ebina, Japan

**CMPR** Collection of Michel Perreau, Paris, France

**CMSB** Collection of Michael Schülke, Museum für Naturkunde Berlin, Germany (J. Frisch)

**CPMG** Collection of Pier Mauro Giachino, Torino, Italy

**MHNG** Muséum d’Histoire Naturelle, Genève, Switzerland (G. Cuccodoro)

**MNHN** Muséum National d’Histoire Naturelle, France, Paris (T. Deuve, A. Taghavian)

**NHRS** Naturhistoriska Riksmuseet, Stockholm, Sweden (J. Bergsten)

**NMPC** Národní muzeum, Prague, Czech Republic (M. Fikáček, J. Hájek)

**NSMT** National Museum of Nature and Science, Tsukuba, Japan (S. Nomura)

The following measurements in millimetres (mm) were made:

**AL** (antennal length): length between the antennal base and the apex

**BTW** (basitarsal width): maximum width of male proximal protarsomere

**EBL** (extended body length): summation of HL, PL, ELL and length of exposed scutellum, preventing the error introduced by exposed or retracted head

**ELL** (elytral length): length between the posterior end of scutellum and the elytral apex

**ELW** (elytral width): widest part of both elytra combined

**EW** (eye width): maximum width of a single compound eye in dorsal view

**HL** (head length): length between the anterior apex of clypeus and the posterior margin of occipital carina along the midline

**HW** (head width): maximum width of head (usually including eyes)

**PL** (pronotal length): length of the pronotum along the midline

**PW** (pronotal width): maximum width of pronotum

**TW** (tibial width): maximum width of male protibia (excluding spines along outer margin etc.)

## Results

### Genus Ptomaphagus

Taxon classificationAnimaliaColeopteraLeiodidae

Hellwig, 1795

#### Distribution.

Holarctic, north Oriental, north Neotropical.

### Subgenus Ptomaphagus

Taxon classificationAnimaliaColeopteraLeiodidae

s. str.

#### Distribution.

Palaearctic, north Oriental.

### Key to species of *Ptomaphagus* Hellwig from eastern Asia

**Table:** 

1	Body length ≥ 4.3 mm (Wang et al. 2016b: Fig. 2A–D); antennomere III much longer than II (Wang et al. 2016b: Fig. 5A); VI subquadrate, length/width = 0.8 (Wang et al. 2016b: Fig. 5A); metathoracic wings absent; aedeagus with median lobe turning to right at apex (Wang et al. 2016b: Fig. 6F–I); spermatheca discoid in distal part (Wang et al. 2016b: Fig. 7A–C); China (Taiwan) (group *yasutoshii*)	**P. (s. str.) yasutoshii Nishikawa**
–	Body length usually ≤ 4.3 mm; antennomere III shorter than or as long as II; VI transverse, length/width ≤ 0.5; metathoracic wings fully developed; aedeagus with median lobe not turning to right at apex; spermatheca not discoid in distal part	**2**
2	Body length ≤ 3.0 mm, except P. (s. str.) masumotoi approaches 3.5 mm; spermatheca sinuous or coiled in distal part (Figs 3F; 5F) (group *nepalensis*)	**3**
–	Body length ≥ 3.5 mm; spermatheca simply curved in distal part (Figs 7F; 9G) (group *sibiricus*)	**5**
3	Elytral apices with sexual dimorphism, rounded in male but acuminate in female (Wang et al. 2016a: Fig. 5G–H); male abdominal ventrite VIII rounded at posterior edge and with a small median notch (Wang et al. 2016a: Fig. 5I); China (Zhejiang), Japan	**P. (s. str.) piccoloi Wang, Růžička, Nishikawa, Perreau & Hayashi**
–	Elytral apices without sexual dimorphism, similarly rounded in both sexes; male abdominal ventrite VIII emarginate at posterior edge	**4**
4	Male basal three protarsomeres less expanded (Fig. 2C); spiculum gastrale of genital segment with about 2/5 of length protruding beyond anterior edge of epipleurite IX (Fig. 2J); aedeagus with the apex of median lobe lanceolate (Fig. 3A, C); Nepal	**P. (s. str.) nepalensis Perreau**
–	Male basal three protarsomeres strongly expanded (Fig. 4C); spiculum gastrale of genital segment with about 1/5 of length protruding beyond anterior edge of epipleurite IX (Fig. 4J); aedeagus with the apex of median lobe oblong (Fig. 5A, C); Thailand	**P. (s. str.) masumotoi Nishikawa**
5	Spermatheca not coiled in proximal part (Wang et al. 2016a: Fig. 4G); China (Taiwan), Japan	**P. (s. str.) kuntzeni Sokolowski**
–	Spermatheca coiled in proximal part (Figs 7F; 9G)	**6**
6	Male abdominal ventrite VIII distinctly emarginate at posterior edge (Fig. 8I); in lateral view, aedeagal median lobe abruptly and strongly bent ventrally in apical part (Fig. 9C); China (Yunnan)	**P. (s. str.) haba sp. n.**
–	Male abdominal ventrite VIII rounded or subtruncate at posterior edge; in lateral view, aedeagal median lobe not abruptly and strongly bent ventrally in apical part	**7**
7	Male abdominal ventrite VIII without a small median notch (Fig. 6I); aedeagus very short and stout (Fig. 7A–B); China (Henan)	**P. (s. str.) funiu sp. n.**
–	Male abdominal ventrite VIII with a small median notch; aedeagus long and slender	**8**
8	Spiculum gastrale of genital segment with about 3/8 of length protruding beyond anterior edge of epipleurite IX (Wang et al. 2016b: Figs 8J; 11J)	**9**
–	Spiculum gastrale of genital segment with about 1/5 of length protruding beyond anterior edge of epipleurite IX (Wang et al. 2016c: Figs 2J; 4J)	**10**
9	Antennomere XI with length/width = 1.9 (Wang et al. 2016b: Fig. 8A); right apicoventral piece of aedeagal median lobe broad (Wang et al. 2016b: Fig. 9H); spermatheca extended leftwards in proximal part (Wang et al. 2016b: Fig. 10B); China (Taiwan)	**P. (s. str.) chenggongi Wang, Nishikawa, Perreau, Růžička & Hayashi**
–	Antennomere XI with length/width = 1.3 (Wang et al. 2016b: Fig. 11A); right apicoventral piece of aedeagal median lobe rather small (Wang et al. 2016b: Fig. 9I); spermatheca not extended leftwards in proximal part (Wang et al. 2016b: Fig. 10C); China (Taiwan)	**P. (s. str.) tingtingae Wang, Nishikawa, Perreau, Růžička & Hayashi**
10	Aedeagus stouter (Wang et al. 2016c: Fig. 5A); right apicoventral piece of median lobe much wider and subpentagonal (Wang et al. 2016c: Fig. 5C); apical half of median lobe thicker in lateral view (Wang et al. 2016c: Fig. 5B); Russia (Far East), South Korea	**P. (s. str.) sibiricus Jeannel**
–	Aedeagus much larger and more slender (Wang et al. 2016c: Fig. 3A); right apicoventral piece of median lobe slenderly lanceolate (Wang et al. 2016c: Fig. 3C); apical half of median lobe much flatter in lateral view (Wang et al. 2016c: Fig. 3B); Russia (Far East)	**P. (s. str.) hayashii Wang, Růžička, Perreau, Nishikawa & Park**

### 

#### Group yasutoshii

Taxon classificationAnimaliaColeopteraLeiodidae

##### Diagnosis.

This group is characterised by the following combination of characters: (1) body length ≥ 4.3 mm; (2) antennomere III much longer than II; VI subquadrate, length/width = 0.8; (3) metathoracic wings absent; (4) aedeagus with median lobe turning to right at apex; (5) spermatheca discoid in distal part. Species included:

P. (s. str.) yasutoshii Nishikawa, 1993 (China (Taiwan))

= P. (s. str.) smetanai Perreau, 1996

#### Group nepalensis

Taxon classificationAnimaliaColeopteraLeiodidae

##### Diagnosis.

This group is characterised by the following combination of characters: (1) body length ≤ 3.0 mm, except P. (s. str.) masumotoi approaches 3.5 mm; (2) antennomere III shorter than or almost as long as II; VI transverse, length/width ≤ 0.5; (3) metathoracic wings fully developed; (4) aedeagus with median lobe not turning to right at apex; (5) spermatheca sinuous or coiled in distal part. Species included:

P. (s. str.) masumotoi Nishikawa, 2011 (Thailand)

P. (s. str.) nepalensis Perreau, 1988 (Nepal)

P. (s. str.) piccoloi Wang, Růžička, Nishikawa, Perreau & Hayashi, 2016 (China (Zhejiang), Japan)

#### Ptomaphagus (s. str.) nepalensis

Taxon classificationAnimaliaColeopteraLeiodidae

Perreau, 1988

**Figure 1.**
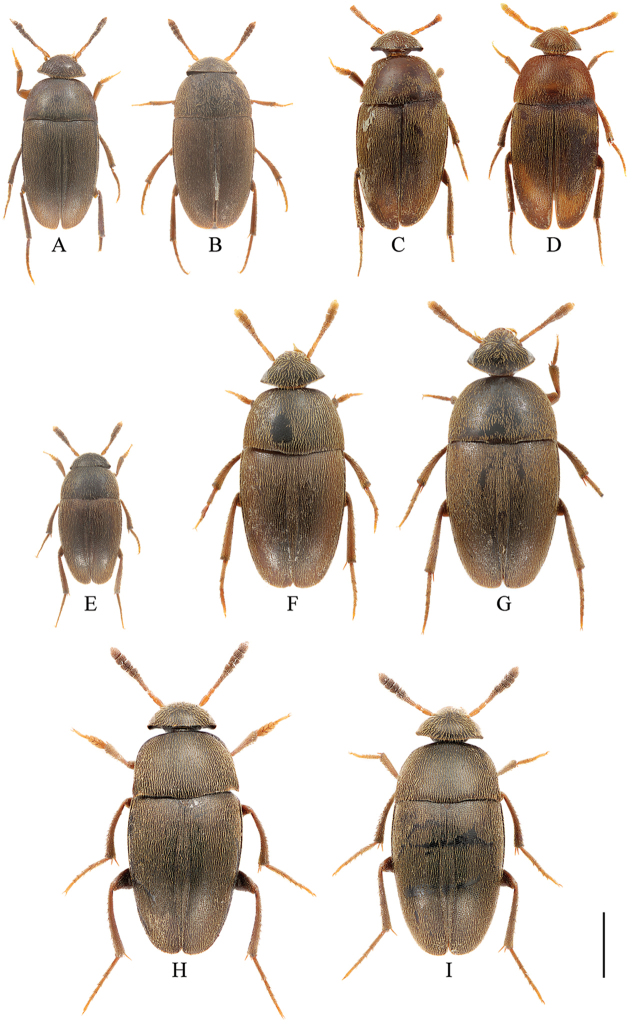
Habitus of *Ptomaphagus* (s. str.) spp. (dorsal view). **A–B** P. (s. str.) nepalensis Perreau, 1988 **A** ♂ (paratype; Nepal) **B** ♀ (paratype; Nepal) **C–D** P. (s. str.) masumotoi Nishikawa, 2011 **C** ♂ (paratype; Thailand) **D** ♀ (holotype; Thailand) **E** P. (s. str.) piccoloi Wang, Růžička, Nishikawa, Perreau & Hayashi, 2016 ♂ (China: Zhejiang) **F–G** P. (s. str.) funiu sp. n. **F** ♂ (holotype; China: Henan) **G** ♀ (paratype; China: Henan) **H–I** P. (s. str.) haba sp. n. **H** ♂ (holotype; China: Yunnan) **I** ♀ (paratype; China: Yunnan). Scale bar: 1 mm.

**Figure 2.**
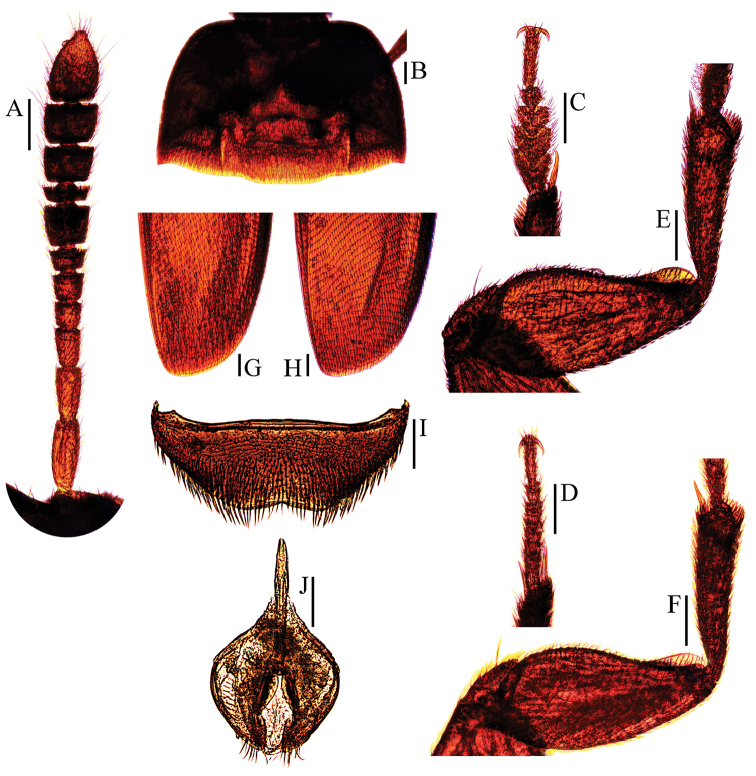
Ptomaphagus (s. str.) nepalensis Perreau, 1988 (♂: paratype; ♀: paratype). **A** antenna ♂ (dorsal view) **B** pronotum ♂ (dorsal view) **C** protarsus ♂ (dorsal view) **D** protarsus ♀ (dorsal view) **E** protibia and profemur ♂ (ventral view) **F** protibia and profemur ♀ (ventral view) **G** elytral apex ♂ (dorsoapical view) **H** elytral apex ♀ (dorsoapical view) **I** ventrite VIII ♂ (ventral view) **J** genital segment ♂ (ventral view). Scale bars: 0.1 mm.

**Figure 3.**
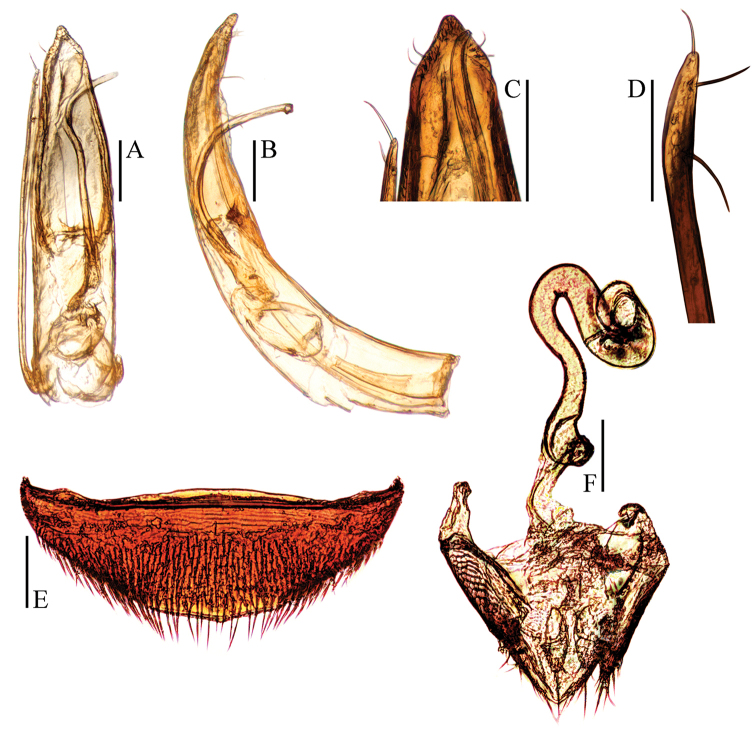
Ptomaphagus (s. str.) nepalensis Perreau, 1988 (♂: paratype; ♀: paratype). **A** aedeagus (dorsal view) **B** aedeagus (lateral view) **C** aedeagal apex (dorsal view) **D** paramere apex (lateral view) **E** ventrite VIII ♀ (ventral view) **F** spermatheca, genital segment and ovipositor (ventral view). Scale bars: 0.1 mm.

Figs 1A–B ; 2A–J ; 3A–F

Ptomaphagus (s. str.) nepalensis Perreau, 1988: 1005 (Ptomaphagus; type locality: Népal, district de Lalitpur, Phulcoki [ca. 27°34'N 085°25'E], 2600 m; MHNG); Perreau, 2000: 363 (Ptomaphagus (s. str.); in catalog); Perreau, 2004: 178 (Ptomaphagus (Ptomaphagus); in catalog); Perreau, 2015: 249 (Ptomaphagus (Ptomaphagus); in catalog).

##### Material examined.

***Type material*. Paratypes**: 1♂, NEPAL, Kath- / mandu District // Phulcoki 2600 m / 20.IV.1982 / A. & Z. Smetana // PARATYPE (MHNG); 1♂, NEPAL Lalitpur / Distr. Phulcoki / 2600 m 16.X.[19]83 / Smetana & Löbl // Ptomaphagus / nepalensis / Perreau 1988 // PARATYPE // MHNG / ENTO / 00003344 (MHNG); 1♀, NEPAL Lalitpur / Distr. Phulcoki / 2600 m 14.X.[19]83 / Smetana & Löbl // PARATYPE // MHNG / ENTO / 00003345 (MHNG); 1♀, NEPAL Lalitpur / Distr. Phulcoki / 2650 m 15.X.[19]83 / Smetana & Löbl // PARATYPE // MHNG / ENTO / 00003346 (MHNG); 1♀, NEPAL Lalitpur / Distr. Phulcoki / 2650 m 14.X.[19]83 / Smetana & Löbl // PTOMAPHAGUS / NEPALENSIS n. sp. / M. PERREAU det. 1987 // PARATYPE // MHNG / ENTO / 00003347 (MHNG); 1♀, NEPAL Lalitpur / Distr. Phulcoki / 2700 m 15.X.[19]83 / Smetana & Löbl // PARATYPE (MHNG); 1♂, NEPAL, Kath- / mandu District // Phulcoki 2600 m / 20.IV.1982 / A. & Z. Smetana // PARATYPE // MHNG / ENTO / 00003343 (MHNG); 1♀, NEPAL, Kath- / mandu District // Phulcoki 2600 m / 22.IV.1982 / A. & Z. Smetana // PARATYPE // MHNG / ENTO / 00003342 (MHNG); 2♂♂, NEPAL: distr. / Kathmandu: Phulcoki / 2500 m, 28–29.IV.[19]84 / Löbl - Smetana (CMPR).

##### Redescription.

*Male*. EBL: 2.9–3.0 mm. Length of different body parts: HL : AL : PL : ELL = 0.5 : 0.9 : 0.7 : 1.6 mm; width: HW : EW : PW : ELW = 0.8 : 0.1 : 1.1 : 1.1 mm. Proportion of antennomeres from base to tip in μm (length × width): 132 × 54, 89 × 50, 69 × 50, 45 × 52, 47 × 60, 38 × 72, 74 × 89, 29 × 90, 67 × 97, 75 × 103, 137 × 94.

Habitus (Fig. 1A) elongated oval, regularly convex and sublustrous. Well pigmented: mostly blackish brown; mouthparts, basal three or four antennomeres and apical half of ultimate antennomere, protarsi, and apex of meso- and metatarsi more or less yellowish. Dorsum continually clothed with fine, recumbent, yellowish pubescence. Insertions of pubescence on dorsal surfaces of pronotum, elytra and femora aligned along transverse striolations; interspace between two striolations glabrous.

Head transverse, HW/HL = 1.5. Clypeofrontal suture absent. Clypeus with anterior margin almost straight. Compound eyes well developed, EW/HW = 0.1. Antennae (Fig. 2A) slender, AL/HW = 1.1; antennomere III shorter than II; VI with length/width = 0.5; XI pear-shape.

Pronotum (Fig. 2B) transverse, widest just before hind angles, PW/PL = 1.5. Sides gently arched, gradually narrowing from posterior to anterior; hind angles slightly projected backwards and subacute. Posterior margin widely but shortly protruded in middle part, emarginate near hind angles.

Elytra oval, widest at about basal 2/7, ELL/EW = 1.4. Sides weakly arched, gradually narrowing from widest part to apices; apices (Fig. 2G) rounded. Sutural striae present. Metathoracic wings fully developed.

Prolegs robust, with basal three protarsomeres (Fig. 2C) expanded: TW/BTW = 1.3. Protibiae (Fig. 2E) expanded towards apex. Profemora broad. Mesotibiae arcuate, mesotarsi simply linear. Metatibiae slender, straight, but relatively short.

Abdominal ventrite VIII (Fig. 2I) distinctly emarginate at posterior edge, and much deeper at median. Spiculum gastrale of genital segment (Fig. 2J) with about 2/5 of length protruding beyond anterior edge of epipleurite IX.

Aedeagus (Fig. 3A) rather long and slender, with median lobe gradually narrowing towards a lanceolate apex and terminated to a widely subrounded knob in dorsal view; opening of genital orifice situated on dorsal surface, deeply cut inwards on preapical left margin of median lobe. Ventral surface of the apex of the median lobe (Fig. 3C) inserted with two ventrally oriented setae on the left side and three ventrally oriented setae on the right side; parameres narrow, reaching about apical 1/7 of median lobe, each apex (Fig. 3D) with two long lateral setae and one shorter apical seta. In lateral view (Fig. 3B), median lobe slender, regularly bent ventrally, and gradually tapering to a round apex. Endophallus with stylus quite slender, a transverse nodule in middle region, a cheliform complex just below base of stylus, and a circular complex in the basal region.

*Female*. Similar to male in general appearance (Fig. 1B), including elytral apices (Fig. 2H), but distinguished by the following characteristics: protarsi (Fig. 2D) simply linear; protibiae (Fig. 2F) narrower; abdominal ventrite VIII (Fig. 3E) almost rounded at posterior edge and slightly protruded at median; genital segment and ovipositor as shown in Fig. 3F; spermatheca (Fig. 3F) sinuous or coiled in distal part, not coiled in proximal part.

##### Distribution.

Nepal.

#### Ptomaphagus (s. str.) masumotoi

Taxon classificationAnimaliaColeopteraLeiodidae

Nishikawa, 2011

**Figure 4.**
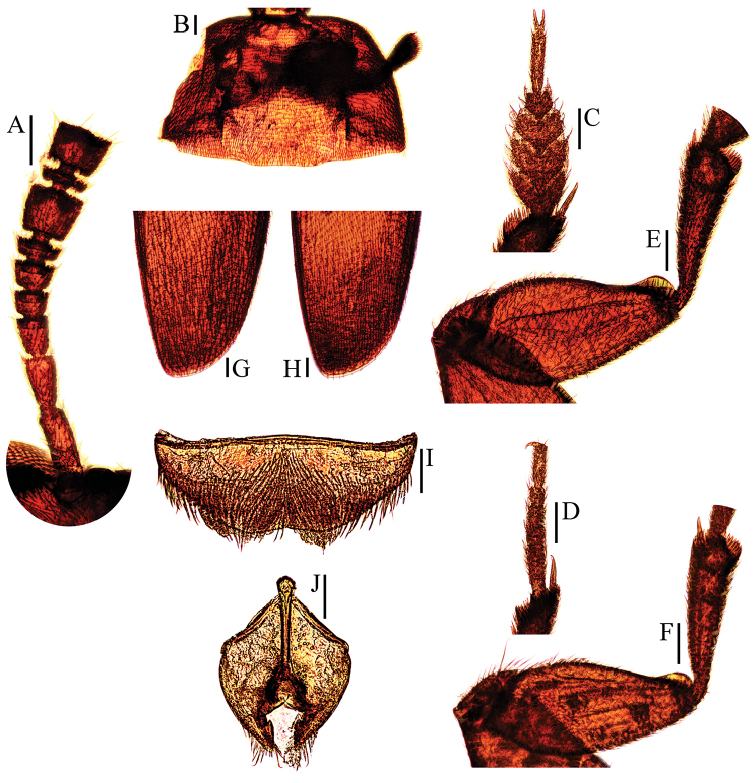
Ptomaphagus (s. str.) masumotoi Nishikawa, 2011 (♂: paratype; ♀: holotype). **A** antenna ♂ (dorsal view) **B** pronotum ♂ (dorsal view) **C** protarsus ♂ (dorsal view) **D** protarsus ♀ (dorsal view) **E** protibia and profemur ♂ (ventral view) **F** protibia and profemur ♀ (ventral view) **G** elytral apex ♂ (dorsoapical view) **H** elytral apex ♀ (dorsoapical view) **I** ventrite VIII ♂ (ventral view) **J** genital segment ♂ (ventral view). Scale bars: 0.1 mm.

**Figure 5.**
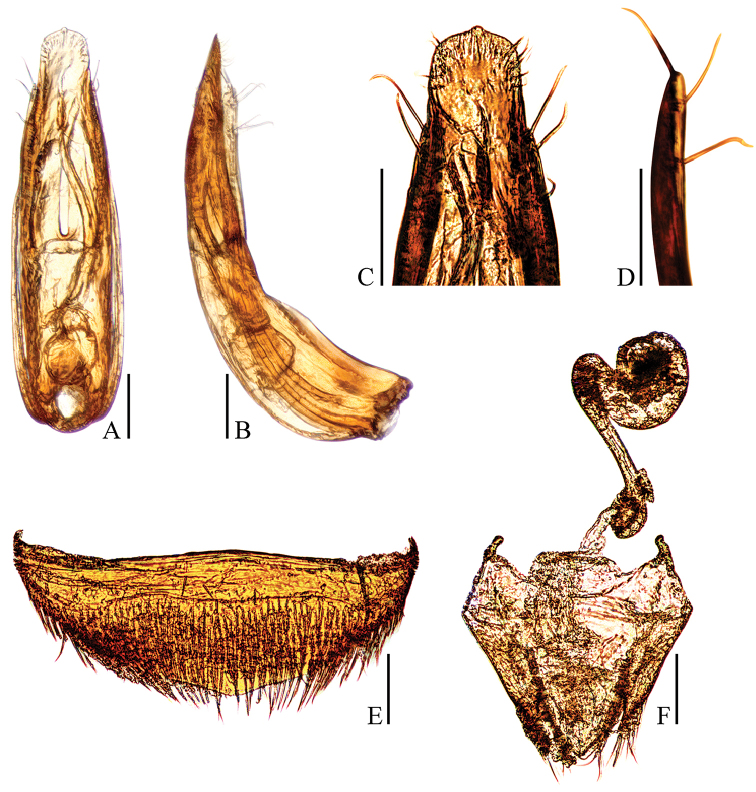
Ptomaphagus (s. str.) masumotoi Nishikawa, 2011 (♂: paratype; ♀: holotype). **A** aedeagus (dorsal view) **B** aedeagus (lateral view) **C** aedeagal apex (ventral view) **D** paramere apex (lateral view) **E** ventrite VIII ♀ (ventral view) **F** spermatheca, genital segment and ovipositor (ventral view). Scale bars: 0.1 mm.

Figs 1C–D ; 4A–J ; 5A–F

Ptomaphagus (s. str.) masumotoi Nishikawa, 2011: 97 (Ptomaphagus (Ptomaphagus); type locality: NW Thailand, Chiang Mai, Doi Inthanon [ca. 18°42'N, 098°59'E], 1750 m; NSMT).

##### Material examined.

***Type material*. Holotype**: ♀, Doi Inthanon / 1750 m, Chiang / Mai, Thailand / 9-XI-1995 / K. MASUMOTO leg. // Holotype / Ptomaphagus (Ptomaphagus) / masumotoi M. Nishikawa, / 2011 / Design. M. Nishikawa, 2011 / # MNC 146Ch2P ♀ (NSMT). **Paratype**: 1♂, same data as holotype except: # MNC 147Ch2P ♂ (NSMT).

##### Redescription.

*Male*. EBL: 3.4 mm. Length of different body parts: HL : AL : PL : ELL = 0.6 : - : 0.8 : 2.0 mm; width: HW : EW : PW : ELW = 0.8 : 0.1 : 1.3 : 1.5 mm. Proportion of antennomeres from base to tip in μm (length × width): 121 × 57, 105 × 62, 71 × 65, 48 × 70, 53 × 82, 35 × 92, 94 × 111, 28 × 101, 83 × 112 (last two antennomeres missing).

Habitus (Fig. 1C) elongated oval, regularly convex and sublustrous. Well pigmented: mostly brown; mouthparts, basal three antennomeres and apical half of ultimate antennomere, protarsi, and apex of meso- and metatarsi more or less paler. Dorsum continually clothed with fine, recumbent, yellowish pubescence. Insertions of pubescence on dorsal surfaces of pronotum, elytra and femora aligned along transverse striolations; interspace between two striolations glabrous.

Head transverse, HW/HL = 1.5. Clypeofrontal suture absent. Clypeus with anterior margin gently rounded. Compound eyes well developed, EW/HW = 0.1. Antennae (Fig. 4A) slender; antennomere III shorter than II; VI with length/width = 0.4; X and XI of holotype missing.

Pronotum (Fig. 4B) transverse, widest around hind angles, PW/PL = 1.6. Sides gently arched, simply narrowing from posterior to anterior; hind angles not projected backwards and bluntly rounded. Posterior margin widely protruded in middle part, emarginate near hind angles.

Elytra oval, widest at about basal 2/7, ELL/EW = 1.4. Sides weakly arched, gradually narrowing from widest part to apices; apices (Fig. 4G) narrowly rounded. Sutural striae present. Metathoracic wings fully developed.

Prolegs robust, with basal three protarsomeres (Fig. 4C) strongly expanded: TW/BTW = 1.0. Protibiae (Fig. 4E) strongly expanded towards apex. Profemora broad. Mesotibiae gently arcuate, mesotarsi simply linear. Metatibiae straight, but relatively short and thick.

Abdominal ventrite VIII (Fig. 4I) emarginate at posterior edge. Spiculum gastrale (Fig. 4J) of genital segment with about 1/5 of length protruding beyond anterior edge of epipleurite IX.

Aedeagus (Fig. 5A) slender, with median lobe gradually narrowing towards an oblong apex and terminated to a shortly rounded knob in dorsal view; opening of genital orifice situated on dorsal surface, deeply cut inwards on preapical left margin of median lobe. Ventral surface of the apex of the median lobe (Fig. 5C) inserted with five ventrally oriented setae on the left side and six ventrally oriented setae on the right side; parameres narrow, reaching about apical 1/7 of median lobe, each apex (Fig. 5D) with two long lateral setae and one similar apical seta. In lateral view (Fig. 5B), median lobe regularly bent ventrally but almost straight in apical half, and gradually tapering to a acuminate apex. Endophallus with stylus quite slender, a transverse nodule in middle region, a cheliform complex just below base of stylus, and a circular complex in the basal region.

*Female*. Similar to male in general appearance (Fig. 1D), including elytral apices (Fig. 4H), but distinguished by the following characteristics: protarsi (Fig. 4D) simply linear; protibiae (Fig. 4F) slightly narrower; abdominal ventrite VIII (Fig. 5E) slightly protruded at median of posterior edge; genital segment and ovipositor as shown in Fig. 5F; spermatheca (Fig. 5F) sinuous or coiled in distal part, not coiled in proximal part.

##### Distribution.

Thailand.

#### Ptomaphagus (s. str.) piccoloi

Taxon classificationAnimaliaColeopteraLeiodidae

Wang, Růžička, Nishikawa, Perreau & Hayashi, 2016

Figs 1E ; 10A, G

##### Material examined.

1♂, CHINA: Zhejiang (CH07-39), / Hangzhou Pref., Tianmu Shan [天目山], 40 / km WNW Linan, water reservoir, / 30°20'56’’N, 119°18'42’’E, 300 / m, plant refuse, litter from rock edges, 17.VI.2007, leg. A. Pütz (CAPE).

##### Remarks.

This species is recorded for the first time from China.

##### Distribution.

China (Zhejiang), Japan.

#### Group sibiricus

Taxon classificationAnimaliaColeopteraLeiodidae

##### Diagnosis.

This group is characterized by the following combination of characters: (1) 3.5 mm ≤ body length ≤ 4.3 mm; (2) antennomere III shorter than or as long as II; VI transverse, length/width ≤ 0.5; (3) metathoracic wings fully developed; (4) aedeagus with median lobe not turning to right at apex; (5) spermatheca simply curved in distal part. Species included:

P. (s. str.) chenggongi Wang, Nishikawa, Perreau, Růžička & Hayashi, 2016 (China (Taiwan))

P. (s. str.) funiu sp. n. (China (Henan))

P. (s. str.) haba sp. n. (China (Yunnan))

P. (s. str.) hayashii Wang, Růžička, Perreau, Nishikawa & Park, 2016 (Russia (Far East))

P. (s. str.) kuntzeni Sokolowski, 1957 (China (Taiwan), Japan)

= P. (s. str.) amamianus Nakane, 1963

P. (s. str.) sibiricus Jeannel, 1934 (Russia (Far East), South Korea)

P. (s. str.) tingtingae Wang, Nishikawa, Perreau, Růžička & Hayashi, 2016 (China (Taiwan))

#### Ptomaphagus (s. str.) funiu sp. n.

Taxon classificationAnimaliaColeopteraLeiodidae

http://zoobank.org/8DA08857-FB58-4688-8E00-38E054A82C3D

**Figure 6.**
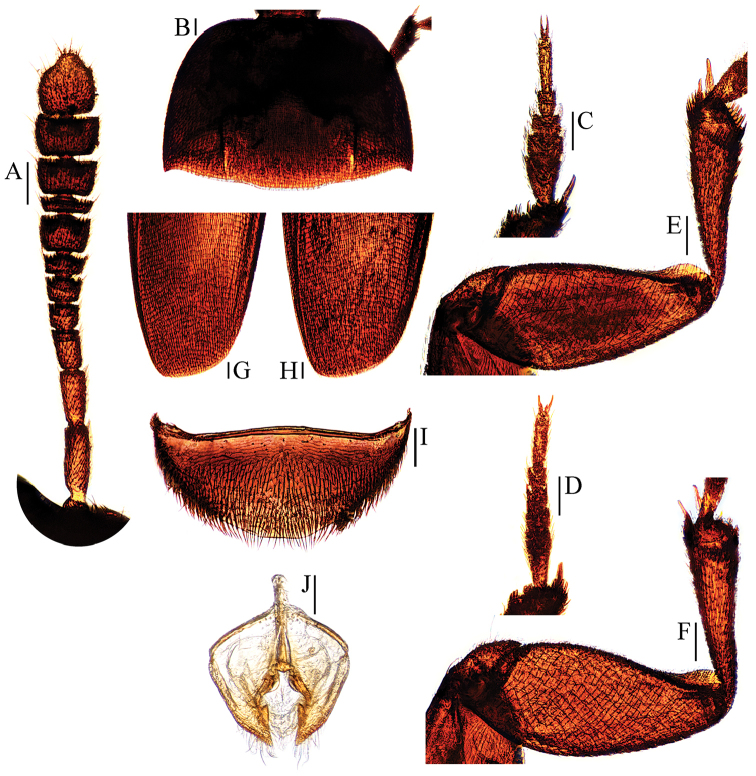
Ptomaphagus (s. str.) funiu sp. n. (♂: paratype; ♀: paratype). **A** antenna ♂ (dorsal view) **B** pronotum ♂ (dorsal view) **C** protarsus ♂ (dorsal view) **D** protarsus ♀ (dorsal view) **E** protibia and profemur ♂ (ventral view) **F** protibia and profemur ♀ (ventral view) **G** elytral apex ♂ (dorsoapical view) **H** elytral apex ♀ (dorsoapical view) **I** ventrite VIII ♂ (ventral view) **J** genital segment ♂ (ventral view). Scale bars: 0.1 mm.

**Figure 7.**
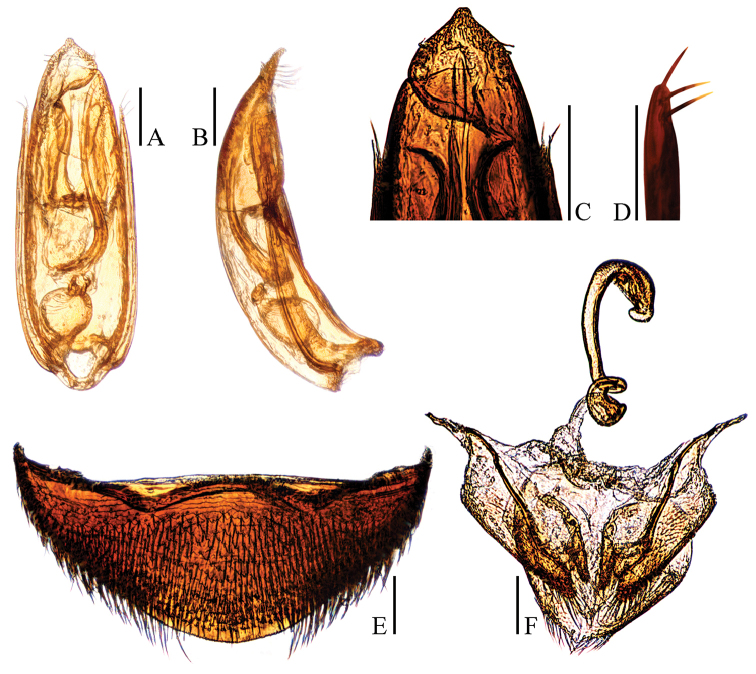
Ptomaphagus (s. str.) funiu sp. n. (♂: paratype; ♀: paratype). **A** aedeagus (dorsal view) **B** aedeagus (lateral view) **C** aedeagal apex (ventral view) **D** paramere apex (lateral view) **E** ventrite VIII ♀ (ventral view) **F** spermatheca, genital segment and ovipositor (ventral view). Scale bars: 0.1 mm.

Figs 1F–G ; 6A–J ; 7A–F

##### Type material.

***Holotype*.** ♂, China, W Henan, 9.–10.VI. / Funiu Shan [伏牛山], 33°31'N, 111°56'E / BAOTIANMAN, 1500–1750 m / Jaroslav Turna leg., 2008 (CPMG). ***Paratypes*.** 1♀, same data as holotype (CJRZ); 1♂, same data as holotype except: 15.V.–5.VI / 2009 (CMPR).

##### Diagnosis.

Aedeagus (Fig. 7A) very short and stout, with median lobe gradually narrowing towards a widely lanceolate apex and terminated to a rounded knob in dorsal view. In lateral view (Fig. 7B), median lobe very thick, gently bent ventrally, and gradually tapering towards a thin apex.

##### Description.

*Male*. EBL: 3.9 mm. Length of different body parts: HL : AL : PL : ELL = 0.6 : 1.0 : 1.1 : 2.1 mm; width: HW : EW : PW : ELW = 1.0 : 0.1 : 1.5 : 1.6 mm. Proportion of antennomeres from base to tip in μm (length × width): 154 × 61, 117 × 61, 87 × 65, 55 × 72, 57 × 87, 46 × 100, 80 × 117, 34 × 124, 77 × 138, 89 × 146, 141 × 131.

Habitus (Fig. 1F) elongated oval, regularly convex and sublustrous. Well pigmented: mostly blackish brown; mouthparts, basal three or four antennomeres and apical half of ultimate antennomere, protarsi, and apex of meso- and metatarsi more or less paler. Dorsum continually clothed with fine, recumbent, yellowish pubescence. Insertions of pubescence on dorsal surfaces of pronotum, elytra and femora aligned along transverse striolations; interspace between two striolations glabrous.

Head quite transverse, HW/HL = 1.7. Clypeofrontal suture absent. Clypeus with anterior margin gently rounded. Compound eyes well developed, EW/HW = 0.1. Antennae (Fig. 6A) slender, AL/HW = 1.0; antennomere III shorter than II; VI with length/width = 0.5; XI pear-shape.

Pronotum (Fig. 6B) transverse, widest directly before hind angles, PW/PL = 1.5. Sides gently arched, narrowing from posterior to anterior; hind angles slightly projected backwards and acute. Posterior margin widely protruded in middle part, emarginate near hind angles.

Elytra oval, widest at about basal 1/5, ELL/EW = 1.3. Sides weakly arched, gradually narrowing from widest part to apices; apices (Fig. 6G) widely rounded. Sutural striae present. Metathoracic wings fully developed.

Prolegs robust, with basal three protarsomeres (Fig. 6C) less expanded: TW/BTW = 1.5. Protibiae (Fig. 6E) expanded towards apex. Profemora broad. Mesotibiae arcuate, mesotarsi simply linear. Metatibiae slender and straight.

Abdominal ventrite VIII (Fig. 6I) simply subrounded at posterior edge. Spiculum gastrale of genital segment (Fig. 6J) with about 1/3 of length protruding beyond anterior edge of epipleurite IX.

Aedeagus (Fig. 7A) very short and stout, with median lobe gradually narrowing towards a widely lanceolate apex and terminated to a rounded knob in dorsal view; opening of genital orifice situated on dorsal surface, deeply cut inwards on preapical left margin of median lobe. Ventral surface of the apex of the median lobe (Fig. 7C) inserted with 5 ventrally oriented setae on the left side and 6 ventrally oriented setae on the right side; parameres narrow, reaching about apical 1/5 of median lobe, each apex (Fig. 7D) with 2 lateral setae and 1 similar apical seta. In lateral view (Fig. 7B), median lobe very thick, gently bent ventrally, and gradually tapering towards a thin apex. Endophallus with stylus quite slender, a subelliptical nodule in middle region, a cheliform complex just below base of stylus, and a circular complex in the basal region.

*Female*. Similar to male in general appearance (Fig. 1G), including elytral apices (Fig. 6H), but distinguished by the following characteristics: protarsi (Fig. 6D) simply linear; protibiae (Fig. 6F) slightly narrower; abdominal ventrite VIII (Fig. 7E) round at posterior edge; genital segment and ovipositor as shown in Fig. 7F; spermatheca (Fig. 7F) curved in distal part, coiled in proximal part, and stem slightly arcuate.

##### Distribution.

China (Funiu Mts. in Henan).

##### Etymology.

The specific epithet is from the Chinese name (in Pinyin) of the type locality “Funiu Shan”, and means “prostrate cow”.

#### Ptomaphagus (s. str.) haba sp. n.

Taxon classificationAnimaliaColeopteraLeiodidae

http://zoobank.org/5A165BE7-0FA5-4439-BFFE-869871DB61C0

**Figure 8.**
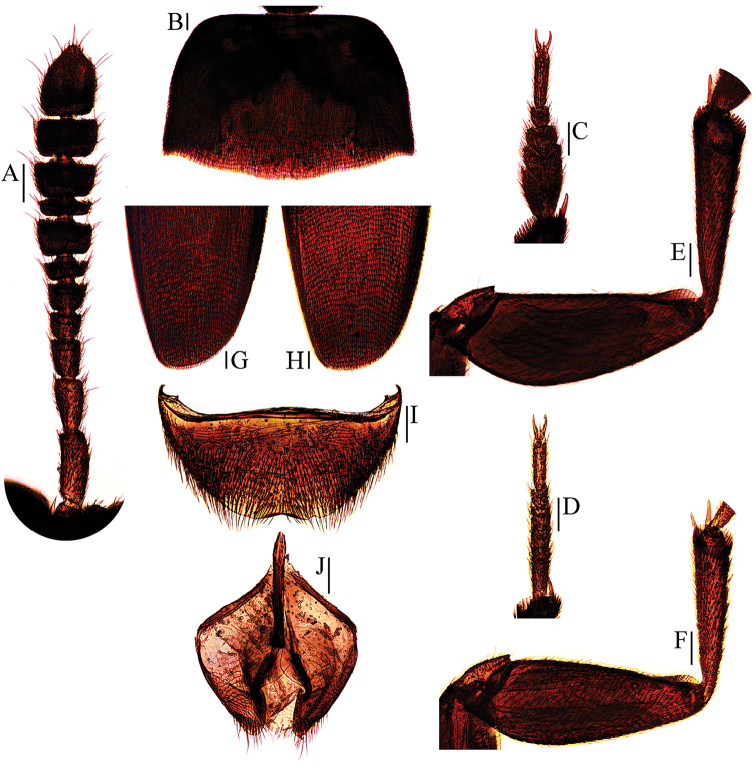
Ptomaphagus (s. str.) haba sp. n. (♂: paratype; ♀: paratype). **A** antenna ♂ (dorsal view) **B** pronotum ♂ (dorsal view) **C** protarsus ♂ (dorsal view) **D** protarsus ♀ (dorsal view) **E** protibia and profemur ♂ (ventral view) **F** protibia and profemur ♀ (ventral view) **G** elytral apex ♂ (dorsoapical view) **H** elytral apex ♀ (dorsoapical view) **I** ventrite VIII ♂ (ventral view) **J** genital segment ♂ (ventral view). Scale bars: 0.1 mm.

**Figure 9.**
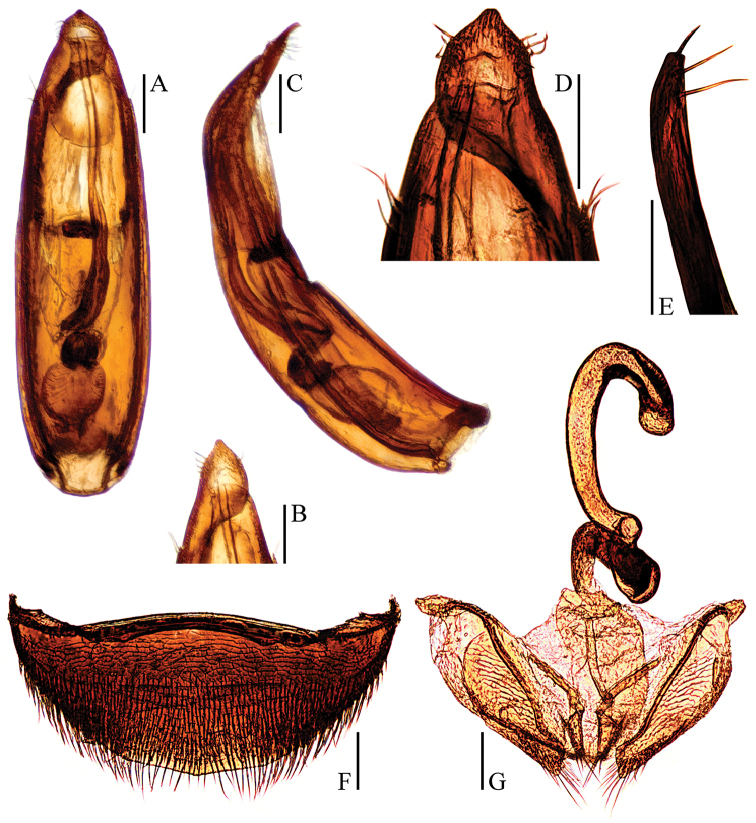
Ptomaphagus (s. str.) haba sp. n. (♂: paratype; ♀: paratype). **A** aedeagus (dorsal view) **B** aedeagal apex (dorsal view) **C** aedeagus (lateral view) **D** aedeagal apex (ventral view) **E** paramere apex (lateral view) **F** ventrite VIII ♀ (ventral view) **G** spermatheca, genital segment and ovipositor (ventral view). Scale bars: 0.1 mm.

Figs 1H–I ; 8A–J ; 9A–G

##### Type material.

***Holotype*.** ♂, CHINA - YUNNAN / HABASHAN [哈巴山] - Habashan Mts. / 12.–16.6.2004, 3500– / 4000 m, 27°19'N, 100°08'E / lgt. Fouquè R.+H. (WGS 84) (NMPC). ***Paratypes*.** 9♂♂, 10♀♀, same data as holotype (1♀ in NMPC, 1♂1♀in BMNH, 1♂1♀ in CCBW, 5♂♂5♀♀ in CJRZ, 1♂1♀ in CMNE, 1♂1♀ in CMPR).

##### Diagnosis.

Aedeagus (Fig. 9A) long and slender, with median lobe gradually narrowing towards a lanceolate apex and terminated to an obtusely rounded knob in dorsal view (Fig. 9B). In lateral view (Fig. 9C), median lobe slender, regularly bent ventrally but abruptly stronger in apical part, and gradually tapering towards a thin apex.

##### Description.

*Male*. EBL: 3.9–4.1 mm (4.0 mm in holotype). Length of different body parts: HL : AL : PL : ELL = 0.6 : 1.1 : 1.0 : 2.2 mm; width: HW : EW : PW : ELW = 1.0 : 0.1 : 1.5 : 1.6 mm. Proportion of antennomeres from base to tip in μm (length × width): 176 × 74, 130 × 75, 88 × 77, 58 × 89, 64 × 101, 38 × 118, 80 × 142, 28 × 136, 84 × 162, 98 × 158, 186 × 140.

Habitus (Fig. 1H) elongated oval, regularly convex and sublustrous. Well pigmented: mostly dark brown to blackish brown; mouthparts, basal two or three antennomeres and apical half of ultimate antennomere, protarsi, and apex of meso- and metatarsi brownish. Dorsum continually clothed with fine, recumbent, yellowish pubescence. Insertions of pubescence on dorsal surfaces of pronotum, elytra and femora aligned along transverse striolations; interspace between two striolations glabrous.

Head quite transverse, HW/HL = 1.6. Clypeofrontal suture absent. Clypeus with round anterior margin. Compound eyes well developed, EW/HW = 0.1. Antennae (Fig. 8A) slender, AL/HW = 1.1; antennomere III shorter than II; VI with length/width = 0.3; XI pear-shape.

Pronotum (Fig. 8B) transverse, widest at hind angles, PW/PL = 1.5. Sides gently arched, narrowing from posterior to anterior, and sensibly constricted above hind angles; hind angles slightly projected backwards and obtusely subrounded. Posterior margin widely protruded in middle part, emarginate near hind angles.

Elytra oval, widest at about basal 1/5, ELL/EW = 1.4. Sides weakly arched, gradually narrowing from widest part to apices; apices (Fig. 8G) rounded. Sutural striae present. Metathoracic wings fully developed.

Prolegs relatively slender, with basal three protarsomeres (Fig. 8C) expanded: TW/BTW = 1.2. Protibiae expanded towards apex. Profemora broad. Mesotibiae arcuate, mesotarsi simply linear. Metatibiae slender and slightly arcuate.

Abdominal ventrite VIII (Fig. 8I) distinctly emarginate at posterior edge. Spiculum gastrale of genital segment (Fig. 8J) with about 1/3 of length protruding beyond anterior edge of epipleurite IX.

Aedeagus (Fig. 9A) long and slender, with median lobe gradually narrowing towards a lanceolate apex and terminated to an obtusely rounded knob in dorsal view (Fig. 9B); opening of genital orifice situated on dorsal surface, deeply cut inwards on preapical left margin of median lobe. Ventral surface of the apex of the median lobe (Fig. 9D) inserted with 6 ventrally oriented setae on both sides; parameres narrow, reaching about apical 1/5 of median lobe, each apex (Fig. 9E) with 2 lateral setae and 1 shorter apical seta. In lateral view (Fig. 9C), median lobe slender, regularly bent ventrally but abruptly stronger in apical part, and gradually tapering towards a thin apex. Endophallus with stylus quite slender, a subelliptical nodule in middle region, a cheliform complex just below base of stylus, and a circular complex in the basal region.

*Female*. Similar to male in general appearance (Fig. 1I), including elytral apices (Fig. 8H), but distinguished by the following characteristics: protarsi (Fig. 8D) simply linear; protibiae (Fig. 8F) narrower; abdominal ventrite VIII (Fig. 9F) almost rounded at posterior edge and slightly protruded at median; genital segment and ovipositor as shown in Fig. 9G; spermatheca (Fig. 9G) curved in distal part, coiled in proximal part, and stem gently arcuate.

##### Distribution.

China (Haba Mts. in Yunnan).

##### Etymology.

The specific epithet is from the Chinese name (in Pinyin) of the type locality “Habashan”, and means “flower of gold” in the Naxi language.

##### Other undescribed species of group *sibiricus*

Certain female specimens of *Ptomaphagus* from eastern Asia are possibly assigned to the right species group, but they cannot be identified at species level without the corresponding male individuals; this problem concerns especially females from the group *sibiricus*. The male aedeagus is the most crucial character for separating species.

**Figure 10.**
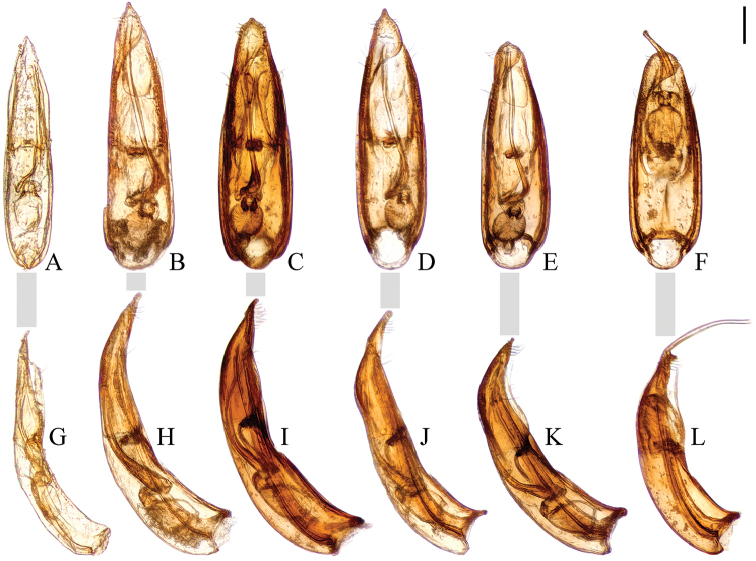
Aedeagi of *Ptomaphagus* (s. str.) spp. (**A–F** dorsal view **G–L** lateral view) **A, G** P. (s. str.) piccoloi Wang, Růžička, Nishikawa, Perreau & Hayashi, 2016 (China: Zhejiang) **B, H** *P.* (s. str.) sp.4 (China: Sichuan) **C, I** *P.* (s. str.) sp.5 (China: Shaanxi) **D, J** *P.* (s. str.) sp.6 (China: Sichuan) **E, K** *P.* (s. str.) sp.7 (China: Shaanxi) **F, L** *P.* (s. str.) sp.8 (China: Shaanxi). Scale bar: 0.1 mm.

The following *Ptomaphagus* species numbered as spp. 1, 2 and 3 have similar spermathecae, all curved in distal part and coiled in proximal part. *Ptomaphagus* spp. 4–8 with only one or two specimens respectively. What is surprising is the syntopic occurrence of four species (spp. 5–8) on a single mountain, Micang Shan (part of the Qinling Mountain Range), even at the same collecting point. For female specimens from the same region, their spermathecae are all curved in the distal part and coiled in the proximal part, similar to each other but with slight differences. Therefore, in consideration of limited specimens and the uncertainly of matching female and male specimens, we refrain from describing these species here and only provide illustrations of their aedeagi in Fig. 10, until such time as more specimens become available from this region.

#### Ptomaphagus

Taxon classificationAnimaliaColeopteraLeiodidae

sp. 1 ♀

##### Material examined.

1♀, N. E. Burma / Kambaiti, 2000 m / 12–17.6.34, Malaise [leg.] // Riksmuseum / Stockholm // Ptomaphagus (s. str.) / kuntzeni Sok. / det. / Szymczakowski 1964 // 9557 / E91 + // NHRS-JLKB / 000027149 (NHRS).

##### Remarks.

Szymczakowski (1964) reported this female specimen as belonging to Ptomaphagus (s. str.) kuntzeni. However, Nishikawa (2011) and Wang et al. (2016a) disputed his identification. After dissecting it, we found that the spermatheca is curved in the distal part and coiled in the proximal part, but P. (s. str.) kuntzeni is so far the only species in the group *sibiricus* in which the spermatheca is not coiled in proximal part. Thus P. (s. str.) kuntzeni is excluded from the list of known fauna of Myanmar.

#### Ptomaphagus

Taxon classificationAnimaliaColeopteraLeiodidae

sp. 2 ♀

##### Material examined.

2♀♀, China, N Henan, 14.VI.–6.VII. / WANGWUSHAN [王屋山], 1650 m / 35°12'N 112°17'E / Jaroslav Turna leg., 2007 (CPMG).

#### Ptomaphagus

Taxon classificationAnimaliaColeopteraLeiodidae

sp. 3 ♀

##### Material examined.

1♀, CHINA: W-Hubei (Daba Shan) / pass E of Mt. Da Shennongjia, / 12 km NW Muyuping [木鱼坪], 31°30'N, / 110°21'E, 22.VII.2001, / leg. M. Schülke [C01-13E] // dry creek vally, mixed deciduous / forest, dead wood, mushrooms, / moss, 1950–2050 m (sifted) [C01-13E] (CMSB); 1♀, China, W Hubei, 20.VI.–12.VII. / MUYUPING [木鱼坪] S.env. ~1300 m / pit fall traps, 31.45N 110.4E / Jaroslav Turna leg., 2003 (CMPR).

#### Ptomaphagus

Taxon classificationAnimaliaColeopteraLeiodidae

sp. 4 ♂

Figs 10B, H

##### Material examined.

1♂, CHINA - NW Sichuan / between Shangliusuo-Luhua / 5 km E of Luhua [芦花镇], 2400 m, shrubs / 7-28.VI.2004, leg. R. Fabbri (CPMG).

#### Ptomaphagus

Taxon classificationAnimaliaColeopteraLeiodidae

sp. 5 ♂

Figs 10C, I

##### Material examined.

2♂♂, China, SW Shaanxi, 21.V.–10.VI. / Micang Shan [米仓山], 32°43'N, 106°34'E / LIPING, for park [forest park], 1700–1850 m / Jaroslav Turna leg., 2009 (CPMG).

#### Ptomaphagus

Taxon classificationAnimaliaColeopteraLeiodidae

sp. 6 ♂

Figs 10D, J

##### Material examined.

1♂, China, N Sichuan, 5.VI.–9.VII. / Micang Shan [米仓山], 1385 m / DABA, 32°40'N 106°55'E / Jaroslav Turna leg., 2007 (CPMG).

#### Ptomaphagus

Taxon classificationAnimaliaColeopteraLeiodidae

sp. 7 ♂

Figs 10E, K

##### Material examined.

1♂, China, SW Shaanxi, 21.V.–10.VI. / Micang Shan [米仓山], 32°43'N, 106°34'E / LIPING, for park [forest park], 1700–1850 m / Jaroslav Turna leg., 2009 (CPMG).

#### Ptomaphagus

Taxon classificationAnimaliaColeopteraLeiodidae

sp. 8 ♂

Figs 10F, L

##### Material examined.

1♂, China, SW Shaanxi, 24.V.–30.VI. / Micang Shan [米仓山], 32°47'N, 106°40'E / LIPING for park [forest park], 1500–1600 m / Jaroslav Turna leg., 2011 (CPMG).

#### Ptomaphagus

Taxon classificationAnimaliaColeopteraLeiodidae

spp. ♀♀

##### Material examined.

7♀♀, China, SW Shaanxi, 21.V.–10.VI. / Micang Shan [米仓山], 32°43'N, 106°34'E / LIPING, for park[forest park] , 1700–1850 m / Jaroslav Turna leg., 2009 (CPMG); 1♀, China, N Sichuan, 5.VI.–9.VII. / Micang Shan [米仓山], 1385 m / DABA, 32°40'N 106°55'E / Jaroslav Turna leg., 2007 (CPMG).

**Figure 11.**
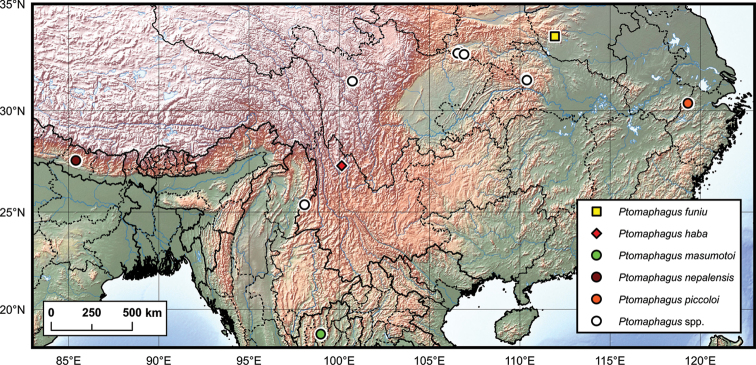
Distribution map of *Ptomaphagus* species from mainland China, Nepal, and Thailand.

## Supplementary Material

XML Treatment for Ptomaphagus

XML Treatment for Ptomaphagus

XML Treatment for yasutoshii

XML Treatment for nepalensis

XML Treatment for Ptomaphagus (s. str.) nepalensis

XML Treatment for Ptomaphagus (s. str.) masumotoi

XML Treatment for Ptomaphagus (s. str.) piccoloi

XML Treatment for sibiricus

XML Treatment for Ptomaphagus (s. str.) funiu

XML Treatment for Ptomaphagus (s. str.) haba

XML Treatment for Ptomaphagus

XML Treatment for Ptomaphagus

XML Treatment for Ptomaphagus

XML Treatment for Ptomaphagus

XML Treatment for Ptomaphagus

XML Treatment for Ptomaphagus

XML Treatment for Ptomaphagus

XML Treatment for Ptomaphagus

XML Treatment for Ptomaphagus
